# Elevated CO_2_ Differentially Mitigated Oxidative Stress Induced by Indium Oxide Nanoparticles in Young and Old Leaves of C3 and C4 Crops

**DOI:** 10.3390/antiox11020308

**Published:** 2022-02-03

**Authors:** Ibrahim I. Shabbaj, Hamada AbdElgawad, Mansour A. Balkhyour, Abdurazag Tammar, Mahmoud M. Y. Madany

**Affiliations:** 1 Department of Environmental Sciences, Faculty of Meteorology, Environment and Arid Land Agriculture, King Abdulaziz University, Jeddah 21441, Saudi Arabia; ishabbaj@kau.edu.sa (I.I.S.); mbalkhyour@kau.edu.sa (M.A.B.); atammar@kau.edu.sa (A.T.); 2 Department of Botany, Faculty of Science, Beni-Suef University, Beni-Suef 62511, Egypt; hamada.abdelgawad@science.bsu.edu.eg; 3 Integrated Molecular Plant Physiology Research, Department of Biology, University of Antwerp, 2020 Antwerp, Belgium; 4 Department of Botany and Microbiology, Faculty of Science, Cairo University, Giza 12613, Egypt; 5 Biology Department, Faculty of Science, Taibah University, Al-Madinah Al-Munawarah 41411, Saudi Arabia

**Keywords:** elevated CO_2_, *Hordeum vulgare*, *Zea maize*, ROS homeostasis, heavy metal, detoxification metabolism

## Abstract

Soil contamination with indium (In) oxide nanoparticles (In_2_O_3_-NPs) threatens plant growth and development. However, their toxicity in plants under ambient (aCO_2_) and elevated (eCO_2_) conditions is scarcely studied. To this end, this study was conducted to investigate In_2_O_3_-NPs toxicity in the young and old leaves of C3 (barley) and C4 (maize) plants and to understand the mechanisms underlying the stress mitigating impact of eCO_2_. Treatment of C3 and C4 plants with In_2_O_3_-NPs significantly reduced growth and photosynthesis, induced oxidative damage (H_2_O_2_, lipid peroxidation), and impaired P and Fe homeostasis, particularly in the young leaves of C4 plants. On the other hand, this phytotoxic hazard was mitigated by eCO_2_ which improved both C3 and C4 growth, decreased In accumulation and increased phosphorus (P) and iron (Fe) uptake, particularly in the young leaves of C4 plants. Moreover, the improved photosynthesis by eCO_2_ accordingly enhanced carbon availability under the challenge of In_2_O_3_-NPs that were directed to the elevated production of metabolites involved in antioxidant and detoxification systems. Our physiological and biochemical analyses implicated the role of the antioxidant defenses, including superoxide dismutase (SOD) in stress mitigation under eCO_2_. This was validated by studying the effect of In_2_O_3_-stress on a transgenic maize line (TG) constitutively overexpressing the *AtFeSOD* gene and its wild type (WT). Although it did not alter In accumulation, the TG plants showed improved growth and photosynthesis and reduced oxidative damage. Overall, this work demonstrated that C3 was more sensitive to In_2_O_3_-NPs stress; however, C4 plants were more responsive to eCO_2_. Moreover, it demonstrated the role of SOD in determining the hazardous effect of In_2_O_3_-NPs.

## 1. Introduction

Soil contamination with heavy metals and their nanoparticles is one of the major constraints altering soil quality [1] and limiting agricultural productivity worldwide [2]. Recently, Indium (In) has become highly accumulated in the soil due to the intensive industry of electronics mainly liquid crystal display and light-emitting diodes as well as solar plates [3]. In air, indium is stable but upon heating it turns into indium oxide (In_2_O_3_). In and its oxide discharge into the environment consequently pose a potential risk to crop growth and development [4]. Many studies have reported that In accumulation in different plant species grown in contaminated soils caused growth inhibition by binding to the cell walls, hence increasing the cell wall rigidity, reducing cell growth, and causing cell rupturing [5,6,7]. Generally, an overdose of metals in the root zone activates the detoxification mechanisms in plants that include metal trapping in apoplast, metal chelation in the cytosol, and metal confinement in the vacuoles [8]. Moreover, indium reduced the uptake and translocation of essential elements such as phosphorus and iron that consequently inhibit plant growth and cause plants to show symptoms of phosphorus deficiency [4].

Similar to its bulk counterpart, indium oxide nanoparticles (In_2_O_3_-NPs) represent an environmental challenge for all living organisms particularly if we know that the world consumption of indium rose to 760 tons in 2019, exhibiting a more than tenfold increase over the last two decades [9]. The toxic effect of In_2_O_3_-NPs on human cells and organs was accentuated in different investigations [10,11]. However, data on the phytotoxic impact of In_2_O_3_ nanoparticles upon plants are scarce and almost non-existent. Therefore, the need is urgent to understand the processes and factors that manipulate the accumulation of In_2_O_3_-NPs and to assess its hazards and risks on plant growth and metabolism.

Metal oxide nanomaterials, in general, adversely affect plant growth and development as they remain on the plant’s surface, causing physical and chemical damage to the plant’s organs [12]. Additionally, these nanomaterials may enter the plant via the root system, making their way to the xylem through the cortex and the pericycle [13]. After accumulation in the plant, metal oxide NPs can interact with the plant either chemically or physically. The chemical interactions include the production of reactive oxygen species (ROS), oxidative damage (e.g., lipid peroxidation), and the interruption of the ion cell membrane transport actfigureivity [13,14], along with the generation of ROS leading to ion leakage and the alteration of the cell metabolism [15]. Obviously, plants developed several detoxification and antioxidant defense mechanisms to cope with phytotoxic effect of metal oxide nanoparticles. Consistently, understanding and exploiting this defense arsenal will improve our ability to control metal oxide NP toxicity. 

Elevated CO_2_ levels are expected to further alter global soil properties and affect the growth and development of agricultural crops. Despite the impact of In and its NPs’ toxicity on global crop yield, little is known on how key crop species handle Al exposure. Indeed, the increment in CO_2_ (eCO_2_) within the physiological range has been proved to improve plant growth by augmenting the photosynthetic carbon metabolism, hence improving plant assimilation [16]. Additionally, a handful of studies have reported that eCO_2_ could mitigate the hazards of different environmental constraints on plant growth and development [16,17,18]. Some investigations attributed the potency of eCO_2_ to alterations in stomatal conductance that consequently enhance water uptake competence [19]. Furthermore, eCO_2_ could alleviate stress by enhancing the plant’s potential to regulate redox homeostasis via manipulating ROS production and trapping [20]. Thus, studying the ability of eCO_2_ to attain plant tolerance under environmental stresses deserves special concern from the environmental scientific community particularly for economically important crops like barley and maize.

C3 and C4 plants differ in their carbon fixation metabolism and consequently differ in their response to environmental stresses. For instance, C3 plants (e.g., barley) and C4 plants (e.g., maize) respond differently to environmental stresses [21]. Unlike C4 plants, C3 plants perform a process known as photorespiration. Photorespiration is considered the main source for reactive oxygen species that obviously affect the cellular redox homeostasis making C3 plants more sensitive to environmental stresses than C4 plants [22]. This leads to the hypothesis that the response of the tested crops to heavy metal NPs such as In_2_O_3_-NPs could be also species-specific. To this end, for the first time, we investigated In_2_O_3_-NPs’ effect on the growth and stress defenses of different growth-stage leaves of C3 (barley) and C4 (maize) crops under current conditions and eCO_2_. Our biochemical analyses implicated the involvement of the superoxide dismutase (SOD) enzyme in In_2_O_3_-NPs’ stress resistance. This role was further validated by investigating the responses of a transgenic maize line (TG) constitutively overexpressing the *AtFeSOD* gene to In_2_O_3_-NPs’ toxicity. Overall, this study could contribute to the improvement of crop quality and productivity and help create future remediation strategies.

## 2. Materials and Methods

Indium oxide nanoparticles (In_2_O_3_) were purchased from American elements, Los Angeles, CA, USA (https://www.americanelements.com/indium-oxide-nanoparticles-nanopowder-1312-43-2, accessed on 4 December 2017). The yellow nano-powder has a specific surface area of 50 m^2^·g^−1^ and purity of 99.99%. In_2_O_3_-NPs are spherical in shape, with an average diameter of less than 50 nm. In_2_O_3_ has a bulk density of 7.18 g/cm^3^ according to the manufacturer’s data. The morphological features were validated by using scanning electron microscope (SEM, JEOL JSM-6510, Tokyo, Japan). To avoid the coarse aggregation of In_2_O_3_ in aqueous solution, NPs were sonicated.

### 2.1. Greenhouse Pot Experiment

This study was performed under conditions that approach environmentally realistic conditions to better understand the real effects of NPs in the environment. Seeds of heavy-metal-stress tolerant plant varieties, i.e., C4 (*Zea maize* L. cv Giza 119) and C3 (*Hordeum vulgare* L. cv Giza 13), were obtained from the Agriculture Research Center, Giza, Egypt. Plant seeds were sterilized by Na-hypochlorite (5% *v*/*v*, 25 min) and 4 seeds of each plant species were sown in PVC tubes (15 cm diameter, 30 cm height) containing sandy soil (96% sand, pH 7.6). The soil initially contained 1.5% carbon, 24 mg nitrate-nitrogen (N), 1.3 mg ammonium-N, and 16 mg phosphorus (P) k^−1^ air dry soil kept at 68% soil water capacity. Plants were grown at a sunlit temperature and in CO_2_-controlled chambers. The interior surface area of each chamber was 1.3 × 1.3 m and the top of the chambers consisted of colorless UV-transparent polycarbonate plate. The temperature was adjusted at 24/19 °C and photosynthetic active radiation (PAR) was measured by a SDEC, type JYP1000 quantum sensor (SDEC, Reignac sur Indre, France). C3 (barley) and C4 (maize) plants were subjected to the following conditions: (1) ambient CO_2_ (aCO_2_, 393 ± 12 ppm); (2) aCO_2_ + In_2_O_3_-NPs (250 mg/kg soil); (3) elevated CO_2_ (eCO_2_, 685 ± 21 ppm); and (4) eCO_2_ + In_2_O_3_-NPs (250 mg/Kg soil). The applied concentration of In_2_O_3_-NPs was selected according to a preliminary experiment, where the effect of several In_2_O_3_-NP concentrations (0, 50, 100, 150, 200, 250, 300, and 400 mg/kg soil) on the growth of barley and maize plants was assessed. A concentration of In_2_O_3_-NPs of 250 mg/kg soil was selected as it reduced the growth of both maize and barley by about 50%. Moreover, eCO_2_ was selected because its concentration, currently 400 ppm, is expected to reach 600–800 ppm before the year 2100 [23]. After 6 weeks of growth, plant samples, i.e., 1st, 2nd, and 3rd leaf (old tissues) and 4th, 6th, and 5th leaf (young tissue), were collected and kept for further analysis. The samples were harvested as biological replicates, with 4 PVC tubes being harvested per each treatment. The fresh and dry weight of roots and shoots was determined, and fresh leaves were kept at −80 °C for biochemical analyses. To investigate if enhancing SOD activity could increase In_2_O_3_-NP tolerance, we grew *FeSOD* overexpressing the maize transgenic line (TG) and its wild type (H99) under In_2_O_3_-NP stress and/or eCO_2_. The TG was induced by overexpressing the *FeSOD* gene from *Arabidopsis thaliana* under the control of the cauliflower mosaic virus 35S promoter; the backcross used was of Pa91 × H99 to the H99 parent [24]. Both TG and its wild type (H99) were grown under similar treatments of In_2_O_3_-NPs and eCO_2_. Seeds were planted on peat potting medium (62% soil water content, Jiffy Products International B.V., the Netherlands). Pots were transferred to the growth chamber under controlled conditions (16-h day/8-h night, 25/18 °C day/night, 300–400 μEm^−2^s^−1^ photosynthetically active radiation provided by high-pressure sodium lamps). Fresh and dry weights were measured, and fresh leaves were kept at −80 °C for biochemical analyses.

### 2.2. Elemental Contents in Plants

Young and old leaves of C3 and C4 plants were washed with deionized water to remove any apoplastic accumulated metal ions. About 250 mg of leaf tissues of C3 and C4 plants were extracted in HNO_3_/H_2_O (5:1) and heavy metals were determined by mass spectrometry, ICP-MS (Finnigan Element XR, Scientific, Bremen, Germany).

### 2.3. Photosynthesis Related Parameters

The light saturated photosynthetic rate was determined with a portable photosynthesis system (LI-6400; LI-COR, Lincoln, The Netherlands). The temperature and CO_2_ concentration in the leaf chambers was kept at 25 ± 0.5 °C and 400 μmol mol^−1^, respectively. All parameters were estimated inside the growth room at noon. The level of chlorophyll a and b and carotenoids was measured in acetone homogenized shoots [25]. RuBisCo activity was measured by a non-radioactive microplate-based assay, which determines the product (3-phosphoglycerate; 3-PGA) in an enzymatic cycle between glycerol-3-phosphate dehydrogenase and glycerol-3-phosphate oxidase [26].

### 2.4. Quantification of Oxidative Damage Markers

The concentrations of H_2_O_2_ were estimated by monitoring the Fe^3+^-xylenol orange complex at 595 nm by the FOX1 method as indicated by the peroxide-mediated oxidation of Fe^2+^, followed by reaction of Fe^3+^ with xylenol orange [27]. The lipid peroxidation level was extracted by homogenized plant tissues in 80% ethanol and then determined by thiobarbituric acid-malondialdehyde (TBA-MDA) reagent [28]. The absorbance was measured at 440, 532, and 600 nm and the content was expressed as nmol g^−1^ FW. Protein carbonyls as oxidative damage markers were measured using the Protein Carbonyl Colorimetric Assay Kit by (Cayman Chemicals Company, Ann Arbor, MI, USA) [29].

### 2.5. Quantification of Antioxidant Parameters

The total antioxidant capacity (FRAP) and antioxidants (phenolics and flavonoids) were extracted in 80% ethanol. After centrifugation (14,000× *g*, 4 °C, 25 min), FRAP assay (0.3 M acetate buffer (pH3.6), 0.01 mM TPTZ in 0.04 mM HCl, and 0.02 M FeCl_3_.6H_2_O) with a Trolox (0 to 650 µM) as a standard was applied [30]. Polyphenols were measured in the supernatant of samples using a Folin–Ciocalteu assay [31]. The flavonoid content was estimated using the modified aluminum chloride method [32].

Ascorbate (AsA) and glutathione (GSH) were measured by HPLC analysis (Shimadzu, Hertogenbosch, the Netherlands). Plant samples were extracted in meta-phosphoric acid (6%, *w*/*v*) after separation on a reversed phase of an HPLC column (Polaris C18-A (100 × 4.6 mm), particle size 3 µm, and 42 °C) [33]. ASC and GSH were detected by diode array detector (DAD) [34].

For antioxidant enzyme activity, proteins were extracted in K-phosphate extraction buffer (50 mM and pH 7.0) containing PVPP (10%, *w*/*v*), Triton X-100 (0.25%, *v*/*v*), and PMSF (1 mM). Peroxidase (POX) was measured by the oxidation of pyrogallol at 430 nm [35], and superoxide dismutase (SOD) enzyme activities and the inhibition of NBT reduction at 560 nm [36]. Dehydr-ASC reductase (DHAR), GSH reductase (GR), ascorbate peroxidase (APX), and monodehydro-ASC reductase (MDHAR) were evaluated spectrophotometrically according to the method of Murshed et al. [37], using 0.05 M MES/KOH). Catalase (CAT) activity was measured by monitoring the rate of decomposition of H_2_O_2_ at 240 nm [38]. The glutathione peroxidase (GPX) activity was assayed by following the reduction of NADPH at 340 nm [39]. The total soluble protein concentration was measured by the Lowry technique [40].

### 2.6. Quantification of Detoxification Related Parameters

GSH-S-transferase was extracted in K-phosphate buffer (50 mM, pH 7.0) containing 0.5 mM CDNB and 1 mM GSH. The activity was estimated according to Mozer et al. [41]. The content of metallothionein (MTC) was electrochemically measured using the differential pulse voltammetry Brdicka reaction according to Diopan et al. [42]. The content of phytochelatins (total thiols-non-protein) was extracted (5% sulfosalicylic acid) and spectrophotometrically measured at 412 nm after mixing with Ellman’s reagent [43].

### 2.7. Determination of Anthocyanins, Phenolics and Flavonoids and the Activity of Related Enzymes

Total anthocyanins were extracted by homogenizing 0.1 g powder frozen in 10 mL acidified methanol (methanol:HCl in 99:1 (*v*/*v*)); the homogenate was incubated at 25 °C for 24 h in the dark then centrifuged at 4000× *g* for 5 min. The anthocyanin content of the extract was quantified by measuring its absorbance at 550 nm and calculating the content using the extinction coefficient 33,000 M^−1^cm^−1^ [44].

Individual phenolic acids and flavonoids were measured (HPLC (SCL-10 AVP, Shimadzu Corporation, Kyoto, Japan). Sample tissues were homogenized in a 4:1 *v*/*v* acetone–water solution. The HPLC system was combined with a column (a Lichrosorb Si-60, 7 μm, 3 × 150 mm) and DAD detector. The mobile phase consisted of 90:10 (*v*/*v*) water–formic acid and 85:10:5 (*v*/*v*/*v*) acetonitrile/water/formic acid at 0.8 mL/min (flow rate); 3,5-dichloro-4-hydroxybenzoic was the internal standard. A calibration curve of the corresponding standard was used to measure the concentration of each compound. The activities of phenylalanine ammonia lyase (PAL) were measured after protein extraction in 200 mM sodium borate buffer at pH 8.8. The activity was monitored by the absorbance of trans-cinnamic acid at 290 nm.

### 2.8. Statistical Analysis

A three-way ANOVA was applied on our results using SPSS (v20.0 software, IBM, Armonk, NY, USA), and significant differences between the means of the parameters (*n* = 4) when comparing the treatments with their respective controls were determined using Fisher’s LSD test (*p* < 0.05). Principal component analysis (PCA) was performed with Origin Lab 9 software (Origin Lab, Northampton, MA, USA).

## 3. Results

### 3.1. eCO_2_ Differentially Enhanced Growth and Photosynthesis of C3- and C4-Plants under the Challenge of In_2_O_3_-NPs

**Figure 1:**
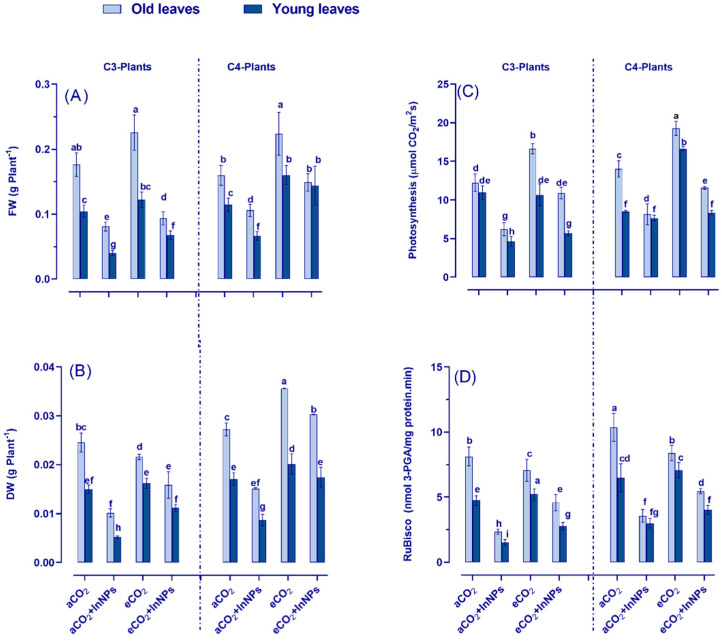
Effect of indium oxide nanoparticles either alone or in combination with eCO_2_ on (**A**) fresh weight (FW); (**B**) dry weight (DW); (**C**) photosynthesis and (**D**) RuBisco activity, of old and young leaves of C3 and C4 plants. Four biological replicates are used to demonstrate each value ± SE. Fisher’s LSD test (*p* < 0.05; *n* = 4) was used to compare the data for each response separately. Different letters indicate significant differences between means in young and old leaves of C3 or C4 plants.

At the growth level, there was a leaf-stage and species-specific response to eCO_2_ and/or In_2_O_3_-NPs. eCO_2_ alone did not significantly affect FW and photosynthesis in C3 (barley) compared to C4 plants (maize) (Figure 1A,C). On the other hand, the fresh and dry biomass as well as the photosynthesis of C3 (barley) plants showed a remarkable reduction in response to In_2_O_3_-NP stress (Figure S1). Moreover, these reductions were more apparent in young leaves than old ones compared to control plants (Figure 1). In more detail, the young leaves of C_3_ plants showed significant reductions in their FW, DW, photosynthesis, and RuBisco enzyme activity by about 40%, 55%, 60%, 80%, respectively, under In_2_O_3_-NP stress. On the other hand, eCO_2_ induced a noticeable recovery in biomass, photosynthesis, and RuBisco activity. Interestingly, C4 plants were more responsive to eCO_2_ than C3 plants. The co-existence of eCO_2_ and In_2_O_3_-NPs caused a striking increment in FW, DW, photosynthesis, and RuBisco activity of C4 young leaves (twofold, 55%, 68%, and twofold, respectively). C_4_ species also responded differently to the treatment with eCO_2_ and In_2_O_3_-NPs (Table S1). Concerning C3 plants, both old and young leaves of barley exhibited partial restoration when treated with eCO_2_. Meanwhile, the treatment of C3 plants with eCO_2_ under In_2_O_3_-NP-contamination conditions led to significant decreases in FW, DW, photosynthetic, and RuBisco activities in their young leaves (~40%, 65%, 30%, and 60% reduction, respectively). Overall, our results indicated that the effects of eCO_2_ and/or In_2_O_3_-NPs were growth-stage and species dependent.

### 3.2. In_2_O_3_-NPs Induced in Accumulation, Particularly in C4 Plants and Reduced P and Fe Uptake

To test if In_2_O_3_-NPs induced In accumulation in target plant leaves, In concentrations were measured in young and old leaves of both C3 and C4 plants. Its levels sharply increased in the old leaves of C3 and C4 but to a greater extent in C4 plants under ambient conditions. In was equally accumulated in young leaves in C3 and C4 plants. Interestingly, eCO_2_ treatment reduced the In uptake of both C3 and C4 plants, and this reduction was higher mainly in the old leaves of C4 (Table 1). In accumulations were reduced by 40% and 23% in C4 and C3 plants, respectively.

Moreover, In can disturb plant mineral nutrition by competition with other nutrients. Thus, concentrations such as phosphorus (P) and iron (Fe) in the old and young leaves of C3 and C4 plants were evaluated in the present study to determine the state of plant P and Fe nutrition (Table 1). The data revealed that In_2_O_3_-NP toxicity reduced the P and Fe content mainly in old leaves of C3 plants under current climate conditions. In contrast, eCO_2_ exposure led to significant improvement in P and Fe concentrations for both plants compared with the corresponding control plants (Table 1). Co-application of eCO_2_ and In_2_O_3_-NPs reduced both element levels mainly in the young leaves of C3, indicating that eCO_2_ was more effective in improving nutrient uptake in the presence of In_2_O_3_-NP stress.

### 3.3. Increased Antioxidant Defense System in C3- and C4-Plants Grown in In_2_O_3_-NP-Polluted Soils Based on Stress Mitigating Impact of eCO_2_

**Figure 2:**
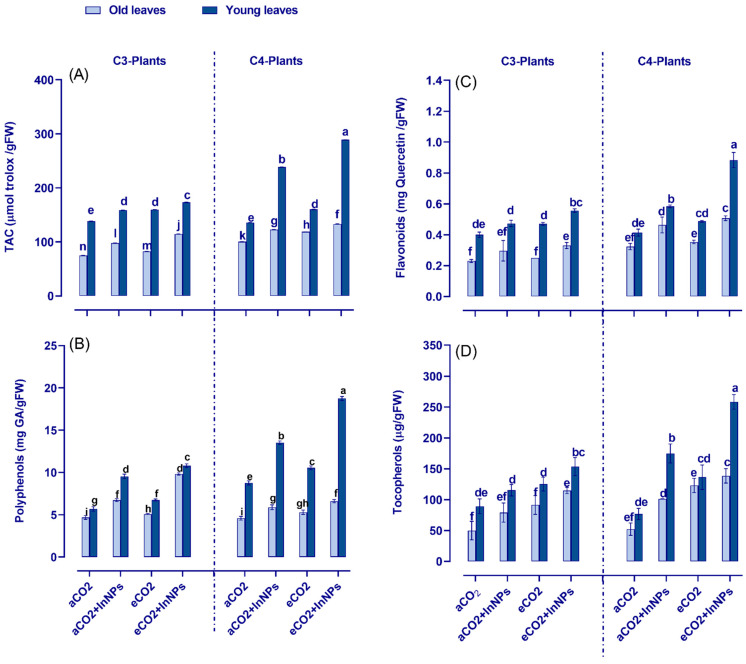
Effect of In_2_O_3_-NPs either alone or in combination with eCO_2_ upon (**A**) total antioxidant capacity (TAC) (**B**) polyphenols, (**C**) flavonoids, and (**D**) tocopherols) of both old and young leaves of C3 and C4 plants. Four biological replicates are used to demonstrate each value. The vertical error bar represents the standard error (SE). Fisher’s LSD test (*p* < 0.05; *n* = 4) was used to compare the data for each response separately. Different letters indicate significant differences between means in young and old leaves of C3 or C4 plants.

To cope with oxidative stress, the plants enhanced their antioxidant capacity to maintain cell viability under In_2_O_3_-NP toxicity. Thus, the total antioxidant capacity (TAC), as well as the molecular antioxidants (i.e., TAC, flavonoids, polyphenols, tocopherols, GSH, and ASC), in C3 and C4 plants (in both old and young leaves) were measured under the effect of eCO_2_ and/or In_2_O_3_-NPs (Figure 2). Individual treatment with In_2_O_3_-NPs caused a remarkable increase in the levels of TAC in the old and young leaves of both C3 and C4 plants. This increase was more pronounced in the young leaves of C4 plants indicating their ability to withstand the stress imposed by In_2_O_3_-NPs (Table S1). Similarly, the molecular antioxidants (tocopherols, GSH, and ASC exhibited a significant accumulation in response to In_2_O_3_-NP treatment (Figure 2). Polyphenols and tocopherols slightly accumulated in the old leaves of both C3 and C4 plants; however, they were highly accumulated in the young leaves of both C3 and C4 plants in response to In_2_O_3_-NPs (Figure 2B,D). Interestingly, eCO_2_ alone caused a noticeable elevation in the TAC of both old and young leaves of C3 and C4 plants as compared with untreated plants (Figure 2A). Contrarily, tocopherols and ASC contents were more enhanced in the old leaves of C3 plants under eCO_2_ (~60% and 100%, respectively), while GSH was induced in the young leaves of C4 plants (Figure 2E). Interestingly, both the old and young leaves of C3 and C4 plants responded differently to the combination of In_2_O_3_-NPs and eCO_2_ (Table S2). Overall, all measured molecular antioxidants (polyphenols, tocopherols, flavonoids, GSH, and ASC) exhibited a remarkable elevation in response to the coexistence of In_2_O_3_-NPs and eCO_2_, especially the young leaves of C4 plants. This mitigative effect of eCO_2_ led to an enhancement in the levels of TAC, particularly in the young leaves of C4 plants in response to eCO_2_ under contamination conditions as compared with contaminated controls grown in ambient CO_2_ conditions.

**Figure 3:**
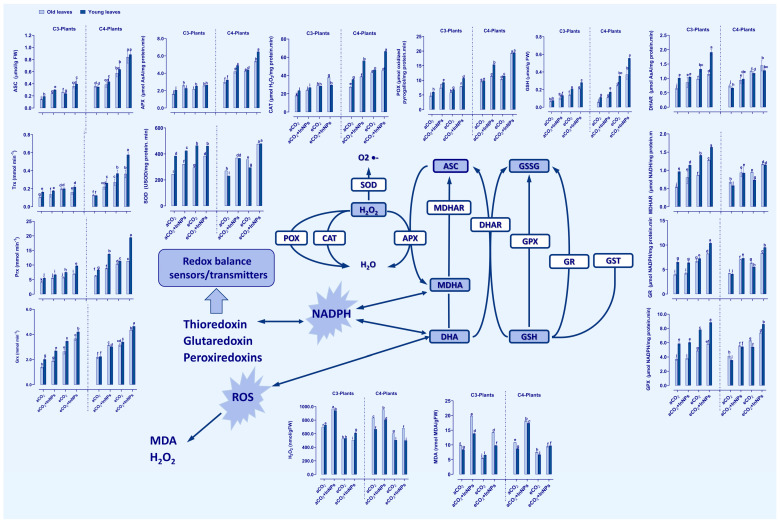
Effect of In2O3-NPs either alone or in combination with eCO2 upon the activities of antioxidant scavenging enzymes as well as the oxidative damage in both old and young leaves of C3 and C4 plants. Four biological replicates are used to demonstrate each value. The vertical error bar represents the standard error (SE). Fisher’s LSD test (*p* < 0.05; *n* = 4) was used to compare the data for each response separately. Different letters indicate significant differences between means in young and old leaves of C3 or C4 plants.

Antioxidant enzymes including those involved in ascorbate/glutathione (ASC/GSH) pool play an indispensable role in ROS homeostasis. In the current study, the enzyme activities of ASC/GSH cycle as well as catalase (CAT), peroxidase (POX), superoxide dismutase (SOD), GSH peroxidase (GXP), peroxiredoxin (Prx), thioredoxin (Trx), and glutaredoxin (Grx) were investigated in both old and young leaves of C3 and C4 plants under the different effects of In_2_O_3_-NPs and/or eCO_2_ (Figure 3). Both old and young leaves of C3 plants have exerted remarkable increases in the activities of POX, SOD, MDHAR (by about 70–100%), CAT, Grx, and Prx (increased by 20–30%). On the other hand, both old and young leaves of C4 plants have exhibited a positive and partially equal response to In_2_O_3_-NP stress on the activities of POX, CAT, APX, DHAR, MDHAR, GPX, Grx, Prx (by about 20–50%), SOD, GR, and Trx (increased by 80–110%) in comparison to control plants. The GR, DHAR, and Trx activities were enhanced only in the old leaves, not in the young ones, while the GPX activity did not show significant changes. Meanwhile, both leaves reacted differently to eCO_2_, whereas CAT, APX, GR, DHAR, and Grx were much more increased in old leaves (by about 80–100%), and Trx was notably increased at the young stage (by about 100%) compared to the control plants. Moreover, the eCO_2_-induced effect was more pronounced on SOD activity only at the young stage, as well as POX activity only at the old stage (Table S2). There were significant differences in the old and young leaves of C4 plants regarding the eCO_2_-induced effect on SOD, which increased by 90% only at the old stage, as well as Trx, which increased dramatically by 200% only at the young stage. In addition, at both stages, the higher levels of CO_2_ have cooperated with In_2_O_3_-NPs to exert much higher increases in most of the measured enzyme activities, particularly Grx, which was dramatically increased by about 200% and 110% in the old and young leaves, respectively. On the other hand, Trx increased dramatically by about 500% in only the young leaves.

### 3.4. In Accumulation under In_2_O_3_-NP Stress Induced Differential Oxidative Damage in C3 and C4 Plants, but Not under eCO_2_ Conditions

Increased lipid peroxidation (MDA) and H_2_O_2_ levels are one of the main oxidative stress indicators for plants [45]. In_2_O_3_-NPs significantly initiate oxidative damage by accumulating MDA and H_2_O_2_ in both the old and young leaves of C3 and C4 plants (Figure 3). The oxidative damage was more pronounced in the old leaves of C3 plants (Figure 3). On the other hand, the eCO_2_ remarkably curbed the oxidative stress by diminishing the accumulation of MDA and H_2_O_2_ in both the old and young leaves of C3 and C4 plants. In barley and maize (C3 and C4 plants), the old leaves and young leaves similarly responded to eCO_2_, which reduced the levels of H_2_O_2_ relative to plants grown under ambient CO_2_ conditions. Meanwhile, stressed old leaves of barley were more responsive to eCO_2_ than young ones in reducing the levels of MDA (Table S2). Moreover, the levels of MDA reduced more clearly in the old leaves than the young leaves of maize (Figure 3). The combination of In_2_O_3_-NPs and eCO_2_ caused further reduction in the levels of H_2_O_2_. This reduction was more obvious in the old leaves than in the young leaves of C3 plants and in young leaves than old ones in C4 plants. On the other hand, the levels of MDA were reduced in both old and young leaves of C3 and C4 plants, with further reduction in old leaves of C4 plants. Overall, C4 plants were more responsive to eCO_2_ either alone or in combination with In_2_O_3_-NPs than C3 in mitigating the oxidative burst caused by the contamination with In_2_O_3_-NPs.

### 3.5. eCO_2_ Improved the Detoxification System of C3- and C4-Plants under the Challange of In_2_O_3_-NPs

**Figure 4:**
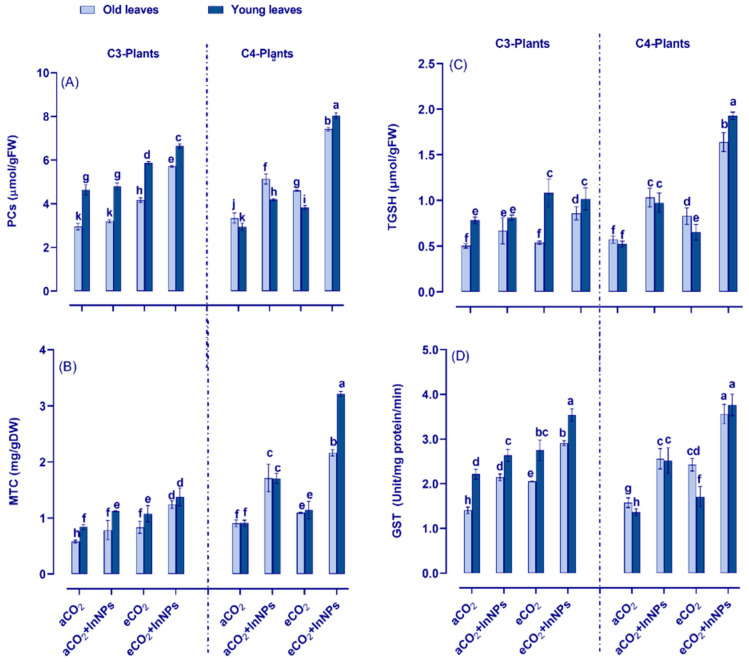
Effect of In_2_O_3_-NPs either alone or in combination with eCO_2_ upon (**A**) phytochelatins (PCs); (**B**) metallothionein (MTC); (**C**) total glutathione (TGSH); (**D**) glutathione transferase (GST) of both old and young leaves of C3 and C4 plants. Four biological replicates are used to demonstrate each value. The vertical error bar represents the standard error (SE). Fisher’s LSD test (*p* < 0.05; *n* = 4) was used to compare the data for each response separately. Different letters indicate significant differences between means in the young and old leaves of C3 or C4 plants.

Higher plants are provided with several strategies to cope with the phytotoxic impact of metal oxide nanoparticles. One of these strategies is the chelation of heavy metals by forming phytochelatins (PCs), metallothioneins (MTC), and total glutathione (tGSH), as well as glutathione transferase (GST). The levels of PCs, MTC, and tGSH as well as the activity of GST were significantly elevated in both the old and young leaves of C3 plants in response to In_2_O_3_-NPs. This enhancing effect was more pronounced in young leaves, especially GST, which had higher activities than the old ones (~90% increase) (Figure 4D). On the other hand, significant increases were observed in PCs, MTC, Tgsh, and GST (by about 80–100%) in stressed C4-plants (at both leaf stages). In addition, the levels of PCs were enhanced only at the old stage, but not at the young one (Figure 4A). Interestingly, the individual treatment of C3 plants with eCO_2_ increased the contents of MTC, PCs, and GST of both old and young leaves. The eCO_2_-induced effect was more obvious in the old leaves, which had higher increments (~80–100% increase) as compared to non-treated plants. It was also observed that the tGSH activity was enhanced only in young leaves, but not in the old one (Figure 4C). Meanwhile, the individual treatment of C4-plants (at both stages) with eCO_2_ has resulted in significant increases in PCs, MTC, Tgsh, and GST, whereby the old stage had higher contents of tGSH and GST than the young stage (increased by 70–100% compared to control). Additionally, the combined treatment with In_2_O_3_-NPs and eCO_2_ has also positively affected the levels of PCs, MTC, GST, and tGSH in the old and young leaves of C3 plants, being higher in the old leaves than the young ones as the increment reached 80–120% in comparison to their counter control plants. Moreover, the PC activity was much more enhanced in the young-stage C4 plants than the old one (increased by about 100% compared to control). The interactive impact imposed by In_2_O_3_-NPs and eCO_2_ on C4-plants has greatly induced the levels of PCs, MTC, tGSH, and GST, whereas PCs and MTC were dramatically increased in the young leaves (by about 300–400%), while tGSH and GST were equally enhanced in both leaves (increased by about 140–200% compared to control). Overall, C4 plants seem to be more responsive than C3 plants, especially to the combined effect of In_2_O_3_-NPs and eCO_2_ on increasing their enzyme activities.

### 3.6. Anthocyanin Metabolism Greatly Improved by Elevated CO_2_ in Both C3- and C4-Plants under the Challenge of In_2_O_3_-NPs

**Figure 5:**
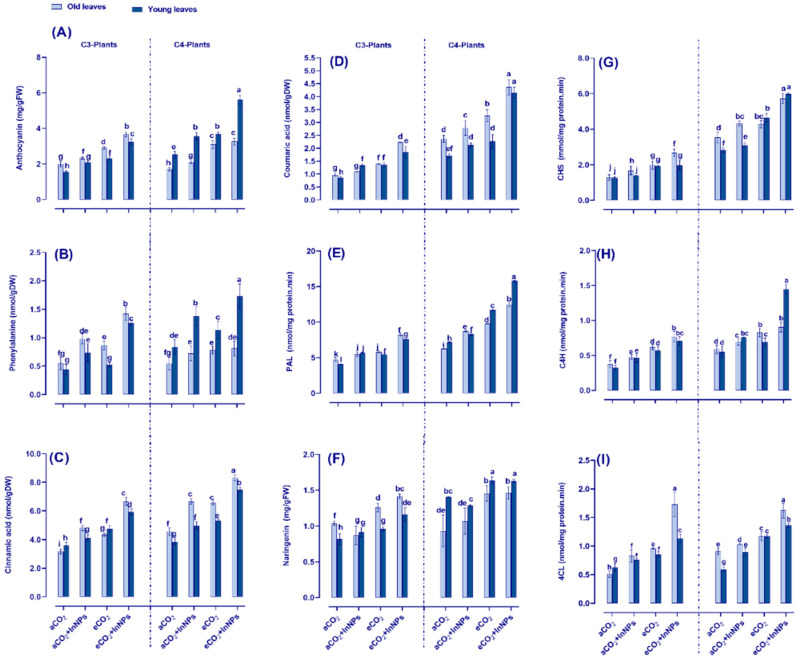
Effect of In_2_O_3_-NPs either alone or in combination with eCO_2_ upon (**A**) anthocyanin, (**B**) phenylalanine, (**C**) cinnamic acid, (**D**) coumaric acid, (**E**) phenylalanine ammonia lyase; PAL, and (**F**) naringenin as well as the activities of (**G**) chalcone synthase; CHS, (**H**) cinnamate-4-hydroxylase; C4H, and (**I**) 4-coumarate CoA ligase; 4CL of both old and young leaves of C3 and C4 plants. Four biological replicates are used to demonstrate each value. The vertical error bar represents the standard error (SE). Fisher’s LSD test (*p* < 0.05; *n* = 4) was used to compare the data for each response separately. Different letters indicate significant differences between means in young and old leaves of C3 or C4 plants.

Our PCA analysis indicated the role of anthocyanins in preventing oxidative damage under In_2_O_3_-NP stress [46]. In this regard, for further assessment of the ROS homeostasis, we shed more light on the anthocyanin metabolism by measuring the levels of anthocyanin metabolism under In_2_O_3_-NPs and/or eCO_2_ (Figure 5). Regarding C3 plants, both old and young leaves exhibited a significant elevation in the contents of anthocyanin, cinnamic acid, and coumaric acid, as well as the activity of (PAL) (~20–40% increase) in response to the individual treatment with In_2_O_3_-NPs. Moreover, phenylalanine exhibited a remarkable increment especially in the stressed young leaves of C4 plants, where the levels were increased by about 68% (Figure 5B). Regarding C4 plants, In_2_O_3_-NP stressed old and young leaves showed increased anthocyanin metabolism. Additionally, the exposure of C3 plants to eCO_2_ enhanced the levels of anthocyanin (~70% increase), phenylalanine, cinnamic acid, coumaric acid, PAL, and naringenin (~20–40% increase). Meanwhile, both old and young leaves of C4 plants responded equally to the individual effect of eCO_2_ on their contents of anthocyanin, cinnamic acid, coumaric acid (increased by about 80–110%), phenylalanine, PAL, and naringenin (by about 40–50%) when compared to the control. Similarly, anthocyanin biosynthetic enzymes (4-coumarate CoA ligase; 4CL, cinnamate-4-hydroxylase; C4H, chalcone synthase; CHS) exhibited a noticeable enhancement in response to eCO_2_ treatment (Figure 5 G–I). By comparing the response of the old and young leaves of C4 plants, cinnamic acid was much more enhanced in old leaves (increased by 100%), while phenylalanine was markedly increased in the young leaves (by about 70%) in reference to their counter control plants. Moreover, the co-treatment of C4 plants with In_2_O_3_-NPs and eCO_2_ led to equal elevations in both old and young plant leaves. Meanwhile, anthocyanins were dramatically increased by about 200% and 400% at the old and young leaves, respectively. Overall, C4 plants better responded to the combined effect of In_2_O_3_-NPs and eCO_2_ on enhancing their anthocyanins and anthocyanin metabolic enzymes.

### 3.7. SOD Overexpression Increased In-Stress Tolerance in Both C3 and C4 Plants

Our study also implicated the role of the SOD enzyme in In_2_O_3_-NP stress mitigation in both leaves of C3 and C4. Thus, further confirming the role of increased activity of SOD enzyme in In_2_O_3_-NP-stress tolerance, we grew the overexpressing *FeSOD* maize transgenic line (TG) and its WT under In_2_O_3_-NPs to investigate the growth, physiology, and biochemical responses of their young and old leaves. At control conditions, overexpressing At*FeSOD* did not significant affect all the investigated parameters, except dry biomass accumulation of old leaves and SOD activity in young leaves (Table 2). The accumulation of In increased by increasing the stress level; nevertheless, TG accumulated less In than WT. Higher accumulation of In induced growth inhibition, where the TG showed lower reduction FW and DW of old and young leaves (Table 2). Consistently, the inhibition of the photosynthesis rate under In_2_O_3_-NPs was more obvious in old leaves of WT (Table 2). Under control conditions, although non-significantly, the concentration of MDA was lower in TG than WT. However, under stress conditions, a significantly lower accumulation of MDA was observed, particularly in old leaves, revealing a better protection of membranes under In_2_O_3_-NP-stress in TG. Under In_2_O_3_-NP stress, TG young and old leaves showed more enhancement in SOD activity than those in WT (Table 2).

### 3.8. Species and Developmental Specific Responses

**Figure 6:**
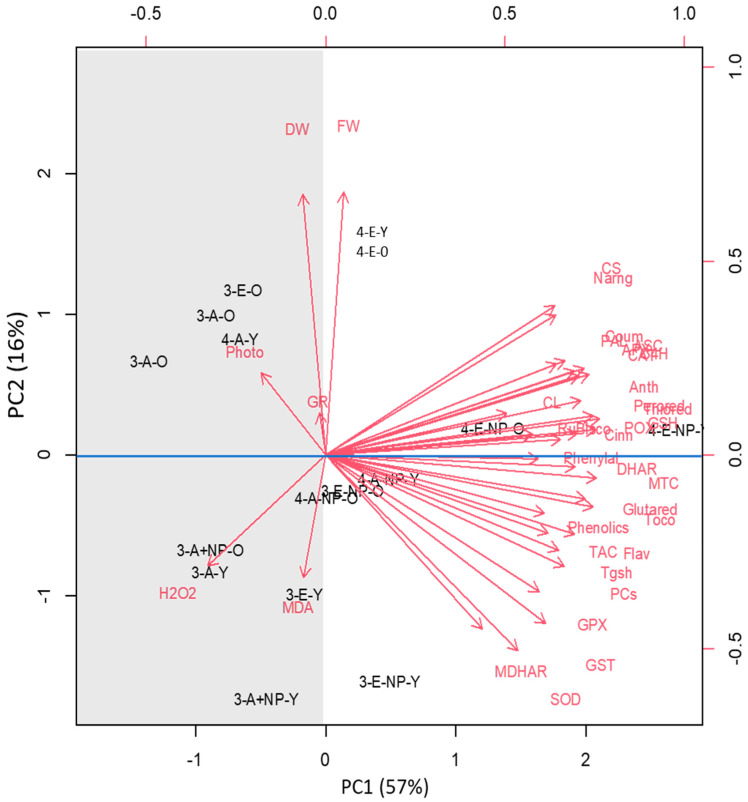
Principal component analysis (PCA) to demonstrate data variability. The arrows demonstrate which variables are most linked with the principal components (PCs). The correlation between variables is determined by the arrow proximity.

Based on the first two components, these plots show standardized scores which explain totally 73% of the data variability (Figure 6). PCA analysis revealed that the responses of C3 plants were separated along the PCA1 (57%) while those of C4 plants were separated along the PCA2 (16%). The distribution pattern was denser for old C3 plants, to the positive side of PC, indicating more related responses in C3 plants under eCO_2_ treatments. Overall PCA showed the age of a leaf affects the plant’s responses to In_2_O_3_-NPs as compared to the effect of eCO_2_. Moreover, cluster analysis of the measured oxidative stress markers and antioxidants showed leaf-stage-specific responses to In_2_O_3_-NPs and eCO_2_. The treatment of old leaves in C3 plants with In_2_O_3_-NPs was allocated one cluster, which highlights the increased levels of dry weight and photosynthesis. The treated young leaves in C4 plants exhibited elevated levels of anthocyanin and anthocyanin metabolic enzymes. Moreover, the young leaves in C3 plants form another cluster that declares a reduction in the oxidative markers (MDA and H_2_O_2_). This was accompanied with an increase in several antioxidant metabolites and enzymes such as GSH, SOD, GPX, and DHAR. Additionally, PCA revealed an apparent separation of responses between elevated CO_2_ from one side and the contamination with In_2_O_3_-NPs on the other. Furthermore, the response of C4 plants to eCO_2_ under contamination conditions can be distinguished from that of C3, especially in young leaves.

## 4. Discussion

This study was conducted to evaluate, for the first time, the phytotoxicity of In_2_O_3_-NPs on old and young leaves under ambient and elevated CO_2_ in two different plant species (C3 (barley) and C4 (maize)). Although many studies have elucidated the impact of eCO_2_ and metal oxide NPs on plants [17,47,48], none of them have addressed the phytotoxicity of In_2_O_3_-NPs as well as the biochemical aspects beneath the ameliorative impact of eCO_2_.

### 4.1. eCO_2_ Alleviated the Growth Reduction and the Oxidative Damage in C3 and C4 Plants Caused by In_2_O_3_-NPs

It is well known that high concentrations of heavy metal NPs dramatically retard the growth and development of different plant species [49]. Our results revealed that In_2_O_3_-NPs greatly affected growth and photosynthetic machinery, particularly in the young leaves of C4 plants. This deleterious effect could be ascribed to the ability of In_2_O_3_-NPs to enter the cell and provoke both molecular and cellular activities [50]. Additionally, this phytotoxic effect could be attributed to the strong binding tendency to the cell walls of plants, the thing that increases cell wall rigidity, reduces cell growth, and causes cell rupturing [6]. Therefore, the accumulation of In may harmfully affect plant growth and metabolism. Moreover, In_2_O_3_-NPs negatively affect the vital processes like cell division, photosynthesis, respiration, and nutrient uptake [51]. In this context, cowpea and rice (C3 plants) showed a noticeable reduction in their growth in response to In accumulation [6,7].On the other hand, the accumulation of In_2_O_3_-NPs can induce limitations to the uptake and translocation of other essential minerals that are required for plant growth. Here, we found that In_2_O_3_-NPs induced In accumulation in both young and old leaves of both C3 and C4 plants, which consequently impaired phosphorous (P) homeostasis. In consistent with our finding, In toxicity altered P uptake and translocation from root-to-shoot by targeting phosphate transporters [4]. This reduction in root-to-shoot translocation in In-treated plants was also explained by phosphate precipitation as In–P complexes. Furthermore, we observed that Fe accumulation in leaves was reduced by In treatment. It is noteworthy that In is chemically similar to Fe [52]; therefore, it can be competitively taken up via the Fe uptake system in plants, causing a reduction in its accumulation.

The reduction in growth can be also explained by the oxidative stress of heavy metals NPs [17,44,47,53]. For instance, Chang et al. [4] reported that In-treated *Arabidopsis thaliana* (C3 plant) exhibited an increase in MDA levels. In agreement, In_2_O_3_-NPs strikingly induced the accumulation of oxidative damage. Increased oxidative damage was embodied in the remarkable increase in the levels of both H_2_O_2_ and MDA, particularly in the old leaves of C3 and C4 plants. A similar accumulation in H_2_O_2_ and MDA was reported in C3 (hordeum and wheat) and C4 (maize) plant species treated with different metal oxide NPs [17,44,48]. It is worth mentioned that heavy metals retard photosynthetic efficiency by diminishing NADPH and ATP utilization in the Calvin cycle, the thing that leads to the overproduction of ROS that severely damage the macromolecules of the cell [54].

On the other hand, our results showed that the adverse effect of In_2_O_3_-NPs was apparently alleviated under the eCO_2_ condition. This mitigative effect was manifested in the restoration of plant biomass and photosynthetic efficiency to almost their normal values (Table 1). Overall, C4 plants (maize) were more responsive to eCO_2_ than C3 plants. In this regard, the elevation of CO_2_, within the physiological threshold, was reported to improve plant growth by boosting photosynthetic carbon metabolism and hence carbohydrate partitioning [16]. These results highlighted the pivotal role of eCO_2_ in protecting important crop plants against such environmental hazards. Consistently with our results, Selim et al. [44] and AbdElgawad et al. [47] found that eCO_2_ greatly improves the growth and photosynthetic machinery of both barley (C3 plants) and maize (C4 plants) under the conditions of As_2_O_3_- and HgO-nanoparticle contamination, respectively.

Indeed, growth induction is accompanied by improving a plant’s ability to manipulate the redox homeostasis including the production and capturing of the reactive oxygen species under stressful conditions [20]. In our study, the coexistence of eCO_2_ with In_2_O_3_-NPs reduced the ROS production (Figure 1). Similarly, eCO_2_ apparently relieved the oxidative damage imposed by other metal oxide NPs such as NiO and HgO nanoparticles on wheat (C3 plants) and maize (C4 plants) via a reduction in the levels of H_2_O_2_ and lipid and protein oxidation [17,48]. The ameliorative effect of eCO_2_ could be ascribed to its ability to reduce the oxygenation reaction of RuBisco [22]. In this regard, eCO_2_ increases the carboxylation rate [16], which consequentially increases carbon assimilation [55]. Additionally, the ameliorative action of eCO_2_ could also attributed to its potential to inhibit the activity of the main enzymes of photorespiration, particularly in old leaves of C3 plants [21].

### 4.2. How Could eCO_2_ Ameliorat the Oxidative Damage Induced by In_2_O_3_-NPs in C3 and C4 Plants?

To add more clarity to the mitigative impact of eCO_2_ on barley and maize that are grown in soil polluted with In_2_O_3_-NPs, we focused on the behavior of detoxification metabolism as well as antioxidant defense systems. Concerning the detoxification metabolism, eCO2 caused a remarkable enhancement in the accumulation of MTC and PCs, as well as GST enzyme activity in C3 and C4 plants with particular enhancement in the young leaves of C4 plants. MTC is a metal binding protein that manipulates the plant metal transport and confinement and GST orchestrates the GSH–metal conjugation [2,56]. Moreover, the accumulatio of PCs including GSH oligomers, will contribute to bind metals and sequester them to the vacuole [57]. In line with our findings, both GST and PCs exhibited a remarkable accumulation in plants subjected to Cd and As [44,56,58,59]. On the other hand, treatment with eCO_2_ not only improved GST activity but also triggered the accumulation of MTC and PCs particularly in old leaves of C3 (barley) and young leaves of C4 (maize) plants. Moreover, exposure to both eCO_2_ and In_2_O_3_-NPs increased all detoxification system components in both C3 and C4 plants especially in the young leaves of C4 plants.

Our PCA analysis suggested that In_2_O_3_-NPs affected the antioxidant defence in both plant species, particularly under eCO_2_. To cope with such an environmental challenge, plants possess different interwoven pathways to maintain safe levels of ROS [60]. These defence pathways involve the production of non-enzymatic antioxidants and enzymatic ROS scavengers [16]. For instance, the ASC/GSH pool underpins the maintenance of redox homeostasis under different environmental challenges [61]. eCO_2_ significantly reduces ROS production and acts as a detoxification system by increasing CO_2_ substrate for RuBisco [20]. eCO_2_-treatment-induced high photosynthesis strengthened carbon input for antioxidant secondary metabolite biosynthesis. Moreover, our results showed a remarkable enhancement in the antioxidant defense system upon treatment with eCO_2_ under ambient and elevated circumstances as well as contamination challenges. This improvement was concomitant with a noticeable elevation in the levels of tocopherols, phenolics and flavonoids in the old leaves of both C3 and C4 plants treated with eCO_2_ under both normal and In_2_O_3_-NP stress conditions. Similarly, Saleh et al. [17] found that eCO_2_ treatment enhanced the accumulation of phenolics, flavonoids, and tocopherols in wheat grown under NiO-NPs. Additionally, the role of CO_2_ in regulating the C and N metabolism cannot be overlooked [62]. This could explain the accumulation of phenolics, tocopherols, and flavonoids in plants grown in a CO_2_-enriched atmosphere, where C and N intermediates and metabolic energy required for their biosynthesis are available [55]. Similar to our findings, Zinta et al. [33] reported that eCO_2_ improved the accumulation of tocopherols and phenolics in *Arabidopsis thaliana* grown under both heat and drought stresses.

Because In_2_O_3_-NPs induced phenolic biosynthesis, the anthocyanin content and metabolism were determined. Anthocyanins are biosynthesized in plants via the phenylpropanoid pathway [63]. In_2_O_3_-NPs as well as eCO_2_ implement an enhancement of the metabolites as well as enzymatic activities of the phenylpropanoid pathway for the biosynthesis of anthocyanin in both barley and maize (Figure 6). Anthocyanins are a class of flavonoids that serve to protect plants under stressful conditions like heavy metal toxicity and their NPs [64]. Similar to our findings, there was upregulation of the anthocyanin metabolism (PAL and CHS) in the cinnamic acid pathway in tissues of barley and maize when exposed to As_2_O_3_-NP [44]. This indicates the importance of this metabolic pathway’s metabolites in heavy-metal-NP stress tolerance. In this regard, anthocyanin can act as a heavy metal chelator [46]. An in vitro assay of the ability of anthocyanins to chelate Cd heavy metal showed a correlation between Cd chelation potency on one side and the contact time and concentration of anthocyanins on the other side [65].

Anthocyanins also have antioxidant properties, and so can defend plant cells from damage via scavenging the stress-induced ROS [44]. Concomitantly, the treatment of *Arabidopsis thaliana* with CeO_2_ NPs caused a significant accumulation in the levels of anthocyanin content [50]. To clarify more, barely tends to accumulate anthocyanins while maize is inclined to accumulate flavonoids and phenolics. In accordance with our results, Selim et al. [44] reported that both barley and maize respond differently when exposed to As_2_O_3_-NPs. Additionally, the tolerance level against heavy metal stress was enhanced in transgenic *Petunia* plants by accumulating anthocyanin in their tissues [66]. Moreover, the coexistence of eCO_2_ with In_2_O_3_-NPs causes additional improvement in the cinnamic acid pathway and hence the accumulation of anthocyanin pigments. A similar finding was reported in two varieties of ginger, which showed a noticeable accumulation in anthocyanin when grown in an atmosphere enriched with high CO_2_ [67].

A principal component analysis revealed a clear and significant separation between C3 and C4 cultivars in terms of antioxidant defenses, including superoxide dismutase (SOD). To confirm the involvement of SOD in In tolerance, we studied the effect of In_2_O_3_-NP-stress on a transgenic maize line (TG) constitutively overexpressing the *AtFeSOD* gene in comparison to its wild type (WT). Overexpressing SOD did not significantly affect the accumulation of In-NPs in either old and young leaves. This suggests that the reduction in growth inhibition and oxidative damage was not in relation to decreased In accumulation in maize leaves. On the other hand, the increased activity of SOD, a vital superoxide anion scavenger, can explain the observed stress mitigation in growth, photosynthesis, and oxidative damage level. Consistently, the expression of transgenic *Arabidopsis FeSOD* in chloroplasts enhanced oxidative stress resistance in tobacco plants by protecting the plasma membranes and PSII [24,68]. In [24], transgenic lines overexpressing *FeSOD* enhanced maize tolerance toward paraquat stress and improved growth under cold stress conditions. Moreover, the overexpression of *AtFeSOD* also enhanced the plant’s ability to increase the activity of other antioxidant enzymes [69].

## 5. Conclusions

**Figure 7:**
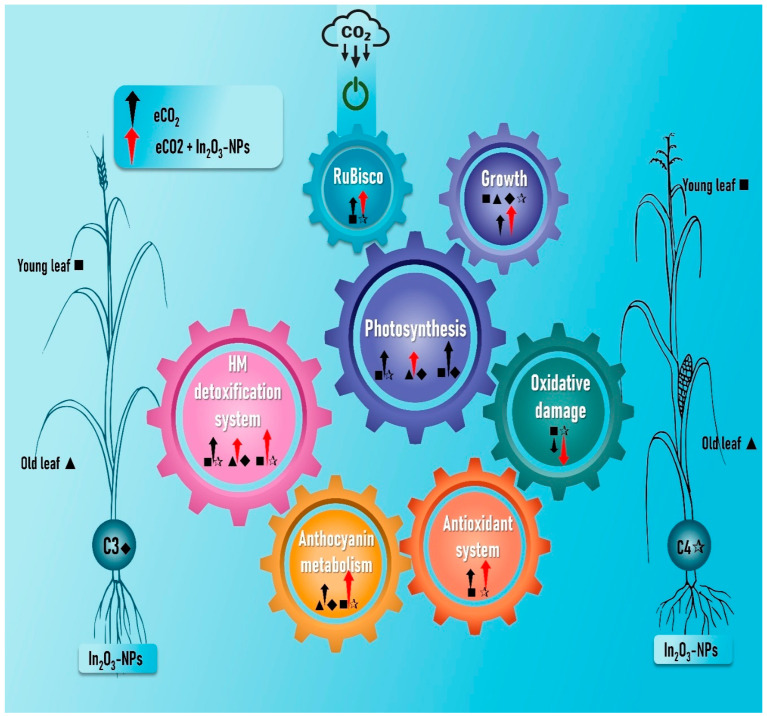
Infographic diagram that summarizes the impact of eCO_2_ and/or In_2_O_3_-NPs upon both old and young leaves of C3 and C4 plants. Black and red arrows indicate either increase or decline due to eCO_2_ or eCO_2_ + In_2_O_3_-NPs, respectively.

Our study was conducted to test the hypothesis that eCO_2_ can mitigate the adverse effect of In_2_O_3_-NPs. Based on our results, elevated CO_2_ can orchestrate the ROS homeostasis and so enhance a plant’s tolerance to contamination with In_2_O_3_-NPs. This enhancement is clearly manifested by a boost in photosynthesis with a concomitant increase in plant biomass particularly in the young leaves of C4 plants under both uncontaminated and contaminated conditions (Figure 7). Additionally, eCO_2_ caused a remarkable reduction in In_2_O_3_-NP-induced oxidative damage by reducing the levels of MDA and H_2_O_2_. Furthermore, extreme CO_2_ significantly enhanced the total antioxidant capacity (TAC) by increasing the accumulation of molecular antioxidants (polyphenols, tocopherols, and flavonoids) in both C3 and C4 plants. The improvement in TAC was accompanied with a noticeable enhancement in the antioxidant scavenging enzymes as well as the AsA/GSH enzymatic pool. The heavy metal detoxification system was also boosted in both C3 and C4 plants grown in an atmosphere enriched with CO_2_ and contaminated with In_2_O_3_-NPs. Moreover, anthocyanins and their related metabolic enzymes also exhibited a significant enhancement in response to eCO_2_, either alone or in combination with In_2_O_3_-NPs. In general, our study provides a new insight in highlighting the pivotal role of eCO_2_ in harnessing the ROS homeostasis in both C3 and C4 crops to withstand the challenge of heavy metal contamination, particularly by In_2_O_3_-NPs.

## Figures and Tables

**Table 1:** Effect of indium oxide nanoparticles and/or elevated CO_2_ (eCO_2_) on the accumulation of Indium (In), Fe, and phosphorus (P) in old and young leaves of C3 and C4 plants. Four biological replicates are used to demonstrate each value ± SE. Fisher’s LSD test (*p* < 0.05; *n* = 4) was used to compare the data for each response separately. Different letters indicate significant differences between means in young and old leaves of C3 or C4 plants.

	C3-Plants							
	Old leaves		Young leaves		Old leaves		Young leaves	
	aCO_2_	aCO_2_ + In_2_O_3_-NPs	aCO_2_	aCO_2_ + In_2_O_3_-NPs	eCO_2_	eCO_2_ + In_2_O_3_-NPs	eCO_2_	eCO_2_ + In_2_O_3_-NPs
In	0 ± 0a	240 ± 12d	0 ± 0a	193 ± 5c	0 ± 0a	171 ± 7bc	0 ± 0a	153 ± 4b
P	4.96 ± 0.6c	2.0 ± 0.12b	3.12 ± 0.1c	1.19 ± 0.06a	5.67 ± 0.5	3.8 ± 0.1bc	3.67 ± 0.1cd	2.25 ± 0.06b
Fe	1.26 ± 0.1bc	0.65 ± 0.02ab	1.02 ± 0.1c	0.47 ± 0.06a	1.27 ± 0.5	0.88 ± 0.1bb	1.17 ± 0.1c	0.75 ± 0.06b
	**C4-Plants**							
	**Old leaves**		**Young leaves**		**Old leaves**		**Young leaves**	
	**aCO_2_**	**aCO_2_ + In_2_O_3_-NPs**	**aCO_2_**	**aCO_2_ + In_2_O_3_-NPs**	**eCO_2_**	**eCO_2_ + In_2_O_3_-NPs**	**eCO_2_**	**eCO_2_ + In_2_O_3_-NPs**
In	0 ± 0a	411 ± 22e	0 ± 0a	226 ± 8c	0 ± 0	295 ± 14d	0 ± 0a	179 ± 3b
P	5.46 ± 0.19d	3.14 ± 0.1b	3.41 ± 0.1c	1.96 ± 0.06a	5.86 ± 0.7d	4 ± 0.3c	4.03 ± 0.06	2.8 ± 0.04ab
Fe	1.51 ± 0.1c	0.92 ± 0.02ab	1.31 ± 0.1b	0.64 ± 0.03a	1.42 ± 0.7d	1.2 ± 0.13b	1.33 ± 0.01c	0.87 ± 0.04bc

**Table 2:** Effect of indium oxide nanoparticles and/or elevated CO_2_ (eCO_2_) on indium (In) accumulation, fresh and dry weight, photosynthesis rate, lipid peroxidation (MDA) and the activity of superoxide dismutase (SOD) in old and young leaves of C3 and C4 plants. Four biological replicates are used to demonstrate each value ± SE. Fisher’s LSD test (*p* < 0.05; *n* = 4) was used to compare the data for each response separately. Different letters indicate significant differences between means in young and old leaves of C3 or C4 plants.

	C3 Plants				C4 Plants			
	Old Leaves		Young Leaves		Old Leaves		Young Leaves	
In	0 ± 0a	387 ± 9.3d	0 ± 0a	215.5 ± 11c	0 ± 0a	352 ± 8.5d	0 ± 0a	178 ± 3.3b
FW	0.24 ± 0.05b	0.13 ± 0.01a	0.17 ± 0.02	0.1 ± 0.01a	0.28 ± 0.01c	0.18 ± 0.01ab	0.22 ± 0.01	0.12 ± 0.01a
DW	0.032 ± 0.003cd	0.018 ± 0.003a	0.022 ± 0.003b	0.013 ± 0.001a	0.038 ± 0.0d	0.024 ± 0.002bc	0.029 ± 0.002c	0.017 ± 0b
Photo	16.4 ± 0.5d	6.8 ± 0.2a	12.2 ± 0.4c	4.5 ± 0.1a	18 ± 0.3d	8.5 ± 0.1b	13 ± 0.3	5.8 ± 0.1a
SOD	122 ± 3.2a	157 ± 1.9b	156 ± 2.6b	201 ± 5.1c	145.4 ± 4.2b	187 ± 2.9c	179.1 ± 2c	224 ± 5.9c
MDA	6.4 ± 0.2ab	9.7 ± 0.9d	5.6 ± 0.4A	7.8 ± 0.1c	5.9 ± 0.2a	7.1 ± 0.9b	5.2 ± 0.3A	6.7 ± 0.9ab

## Data Availability

The data is contained within the article and supplementary material.

## Supplementary Materials

The following are available online at https://www.mdpi.com/article/10.3390/antiox11020308/s1, Figure S1: A photograph that illustrates the differential effects of eC2 either lonely or in combination with In2O3-NPs upon the biomass of C3 and C4 plants, Table S1: A three-way ANOVA for the effect of treatment (Trt.) with eCO2, the plant species (Sp.) and the stage of plant leaf (St.) as well as their interaction on the biomass and photosynthesis as well as the molecular antioxidants and the oxidative markers (numbers represent F values; ns = non-significant; * = *p* < 0.05; ** = *p* < 0.01; *** = *p* < 0.001, **** = *p* < 0.0001). Table S2: A three-way ANOVA for the effect of treatment (Trt.) with eCO2, the plant species (Sp.) and the stage of plant leaf (St.) as well as their interaction on the ascorbate/glutathione biosynthetic pool as well as detoxification system and anthocyanin metabolism (numbers represent F values; ns = non-significant; * = *p* < 0.05; ** = *p* < 0.01; *** = *p* < 0.001, **** = *p* < 0.0001)
