# Supplementary material for: Elevated CO2 Differentially Mitigated Oxidative Stress Induced by Indium Oxide Nanoparticles in Young and Old Leaves of C3 and C4 Crops
Source: Antioxidants (Basel). 2022 Feb 3;11(2):308. doi: 10.3390/antiox11020308 (PMC8868301; doi:10.3390/antiox11020308)

Supplementary tables

Table S1

A three-way ANOVA for the effect of treatment (Trt.) with eCO2, the plant species (Sp.) and the stage of plant leaf (St.) as well as their interaction on the biomass and photosynthesis as well as the molecular antioxidants and the oxidative markers (numbers represent F values; ns= non-significant; *= P < 0.05; ** = P < 0.01; *** = P < 0.001, **** = P < 0.0001)

**Dependent variables**	**Independent variables**						
	**Trt**	**St.**	**Sp.**	**Trt.*St.**	**Trt.*Sp.**	**St.*Sp.**	**Trt.*St.*Sp.**
**Fresh weight**	58.3****	19.45***	260.5****	5.78**	22.17***	13.03*	1.67 ns
**Dry weight**	116.8****	159.2****	23.69****	8.63***	16.14****	16.19***	2.710ns
**Photosynthesis**	84.26****	40.25****	8.14**	3.88*	2.75ns	5.22*	2.10ns
**RuBisco**	133.1***	58.69****	10.50**	1.82ns	6.30***	1.00ns	5.06**
**H_2O_2**	42.25****	48.51****	53.40****	188.2****	1010****	1148****	606.5****
**MDA**	868.4****	137.5****	1.086 ns	221.1****	287.4****	362.3****	352.1****
**TAC**	24205****	17315****	41738****	52630****	53543****	36653****	78152****
**Polyphenols**	1552****	5112****	891.5****	203.1****	10.49****	2080****	222.4****
**Tocopherols**	125.2****	181.3****	70.31****	11..93****	16.55****	8.185**	10.91****
**Flavonoids**	305.9****	477.4****	0.000 ns	64.53****	0.000 ns	0.000 ns	2.119 ns

Table S2

A three-way ANOVA for the effect of treatment (Trt.) with eCO2, the plant species (Sp.) and the stage of plant leaf (St.) as well as their interaction on the ascorbate/glutathione biosynthetic pool as well as detoxification system and anthocyanin metabolism (numbers represent F values; ns= non-significant; *= P < 0.05; ** = P < 0.01; *** = P < 0.001, **** = P < 0.0001)

	Independent variables						
**Dependent variables**	**Trt.**	**St.**	**Sp.**	**Trt.*St.**	**Trt.*Sp.**	**St.*SP.**	**Trt.*St.*Sp.**
**ASC**	115.8****	5.524*	416.7****	0.377^{ns}	29.99****	0.0295^{ns}	1.208
**GSH**	597.2****	218.5****	355.2****	18.26****	94.03****	34.97****	3.47*
**APX**	558.4****	120.9****	4836****	7.931****	182.7***	42.90****	3379****
**DHAR**	111.6****	38.86****	4.043^{ns}	2.155^{ns}	4.052*	77.41****	8.732***
**MDHAR**	153.3****	64.96****	86.14****	0.0022^{ns}	9.686***	150.1****	5.521**
**GR**	1139****	337.7****	8.785**	51.59****	160.7****	261.6****	11.38****
**POX**	3200****	876.1****	1297****	73.35****	647.2****	11.08**	87.02****
**CAT**	2499****	1626****	1347****	1513****	3103****	1687****	4028****
**SOD**	18288****	9176****	1088****	3713****	2835****	2096****	12027****
**GPX**	851.5****	1.012^{ns}	649.7****	65.47****	36.12****	671.9****	19.50****
**Grx**	773.3****	143.7****	245.2****	2.041^{ns}	11.38****	77.18****	4.051*
**Prx**	1832****	2716****	7340****	287.9****	307.5****	424.4****	161.8****
**Trx**	129.7****	254.4****	71.88****	63.49****	10.88****	9.55**	10.52****
**PCs**	1424****	192.4****	23.06****	6.339**	201.3****	547.5****	43.91**
**MTC**	364.89***	463.78****	136.89**	634.89****	1275.1****	349.3***	125.78**
**tGSH**	174.2****	32.66****	90.42****	2.51^{ns}	77.61****	32.12****	12.46****
**GST**	243.8****	0.195^{ns}	32.49****	15.86****	3.452*	78.36****	5.55*
**Anthocyanin**	378.5****	246.6****	104.3****	9.941****	24.49****	393.7****	17.70****
**Phenylalanine**	69.71****	22.97****	16.74***	5.45**	4.278*	117.2****	4.088*
**Cinnamic acids**	136.45***	456.9****	567.5**	234.7****	59.89***	47.87**	158.8***
**PAL**	2297****	7354****	119.4****	357.7****	48.29****	342.1****	97.67****
**Naringenin**	356.8****	942.7****	463.7****	185.8*	163.89****	57.89**	327.7***
**CHS**	136.89****	237.56****	8934.89****	694.56****	223.6****	234.9****	463.9****
**C4H**	972.13****	379.19****	731.84****	896.72****	612.91****	982.15****	138.45*
**4CL**	179.9****	50.07****	51.60****	3.2085*	12.60****	0.101****	11.16^{ns}

Supplementary Figure

Figure S1

A photograph that illustrates the differential effects of eC2 either lonely or in combination with In2O3-NPs upon the biomass of C3 and C4 plants.

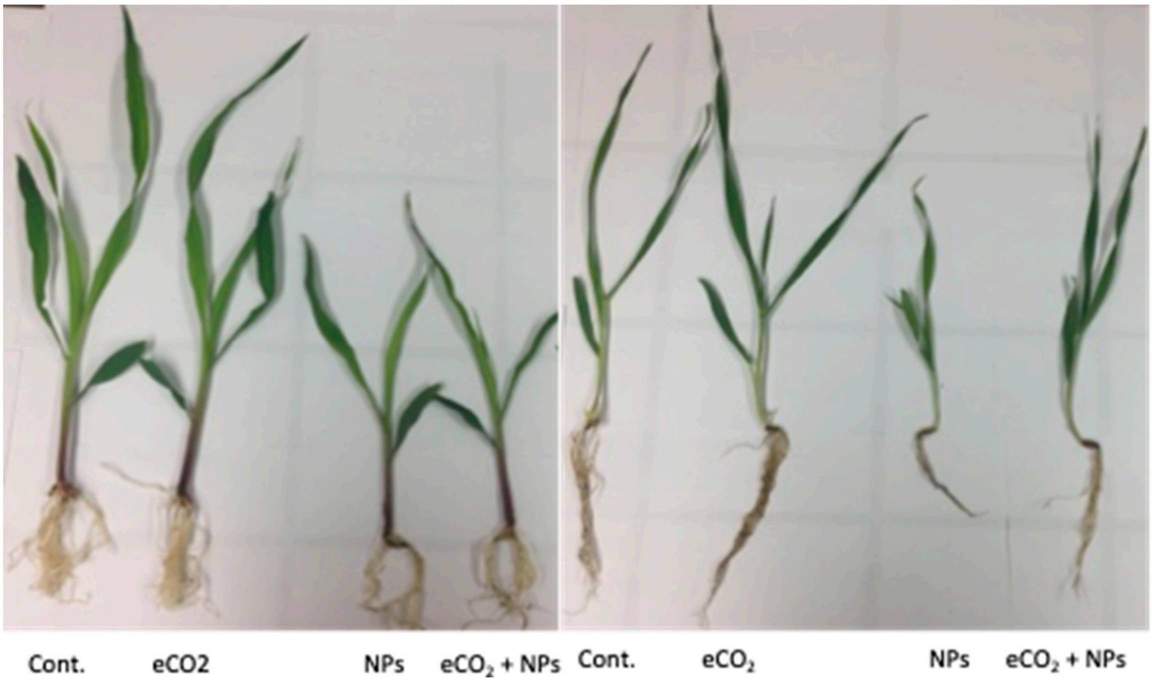

Supplement: Supplementary file 1 [file antioxidants-11-00308-s001.zip › antioxidants-1573413-supplementary.pdf]
